#  Treatment approaches to Motor Speech Disorders: A step towards Evidence Based Practice

**DOI:** 10.12669/pjms.40.3.8096

**Published:** 2024

**Authors:** Anum Ashraf, Nazia Mumtaz, Ghulam Saqulain

**Affiliations:** 1 Anum Ashraf, Senior Lecturer, Faculty of Rehab & Allied Health Sciences, Riphah International University; 2 Nazia Mumtaz, PhD Head of Department of Speech Language Pathology, Faculty of Rehab & Allied Health Sciences, Riphah International University; 3 Dr. Ghulam Saqulain, FCPS (Otorhinolaryngology) HOD & Professor of Otolaryngology, Deputy Dean, Capital Hospital PGMI, Islamabad - Pakistan

**Keywords:** Apraxia, Dysarthria, Motor Speech Disorders, Quality of life, Speech language pathologist

## Abstract

Motor Speech Disorders is an umbrella term for a set of separate dysfunctions of speech outcome associated with neurological disorders. Motor speech disorders (MSD) are classified as Speech Motor delay (SMD), Childhood dysarthria (CD), Childhood Apraxia of Speech (CAS), and Concurrent CD and CAS. The incidence and prevalence of MSD in population is uncertain. A research gap exists, making evidence-based practice questionable as regards intervention for MSD and is an area of research.

Hence, current narrative review was conducted to review and highlight treatment of MSD since evidence-based treatment approach may benefit patient even years after a brain lesion. To achieve this objective literature search was conducted using search engines and data bases including google, google scholar, web of science & PubMed from 1998 to 2023 with keywords “motor speech disorder, dysarthria, apraxia, speech motor delay and combinations of these words with English language and no other limitations. Our search revealed 170 articles, news, publications of which 34 were used for review (Fig.1).

## INTRODUCTION

Motor speech Disorders (MSD) encompass a set of neurological disorders of speech which can be classified on the basis of anatomy and physiology, neuropathological features, acoustic-phonetic factors, or copies of speech output.^1^ MSD are classified as Speech Motor delay (SMD), Childhood dysarthria (CD), Childhood Apraxia of Speech (CAS), and Concurrent CD and CAS.^2^ The incidence and prevalence of motor speech disorders (MSD) in population is uncertain and four types of MSD have been reported in epidemiological research conducted in US, UK and Australia as SMD 4/1000, CD 1/1000 and CAS 1/1000 in children.^3^

**Fig.1:**
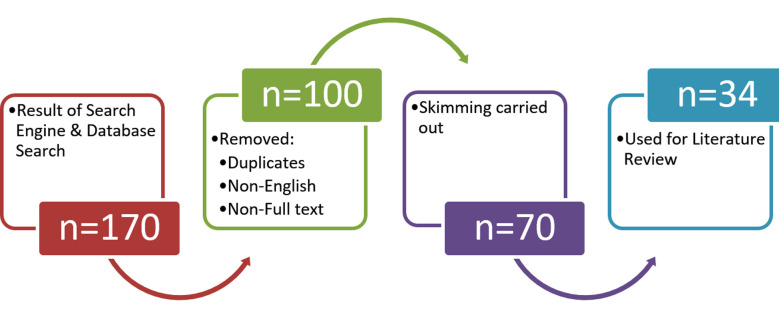
Schematic representation of literature search.

Dysarthria is grouped as MSD resulting from neurological causes leading to weakness, sluggishness, and incoordination of oral musculature consequently presenting issues of respiration, phonation, articulation, resonance and prosody. Dysarthria results in slurring due to poor respiratory control, problems in phonation, facial musculature, restricted tongue movements, decreased labial and lingual movements, and disrupts speech accuracy and speed leading to communication discrepancies thus, effecting the quality of life (Qol).^4^ The identification of MSD depends upon neurologist’s effort to localize disease and establish neurological diagnosis. Speech language pathologists (SLPs) further classify the kind of dysarthria by matching its clinical features to the type of dysarthria. A research gap exists, making evidence-based practice questionable as regards clinical features and intervention for dysarthria resulting from stroke.^5^

While in apraxia, there is incapability to perform purposeful movement in spite of normal muscular tone and coordination. It is classified as limb, oral and verbal apraxia. Focusing on the two major motor speech issues, oral and verbal apraxia, both are confused together but the patients with oral apraxia cannot coordinate oral motor movements. Whereas difficulty in motor programming and production is known as verbal apraxia.^6^ A study of treatment strategies from 2004 to 2012 showed support for articulatory-kinematic as well as rate-rhythm approach for these cases.^7^ Also motor learning principles in intervention for CAS has limited evidence, but is encouraging and the principles and concept provide important framework to further find optimal treatment strategy and can be marked as an significant area of research.^8^ Speech Motor Delay (SMD) is a childhood disorder which has been proposed rather recently. It is evident from the speech which is unstable and lacks precision; prosody and voice which cannot be categorized as dysarthria or apraxia of childhood can be labelled as SMD which can persist into late adolescence in more than 20% of the concerned population.^9^

## METHOD

Keeping in view the high prevalence, research gap as regards treatment of MSD^5^,^8^, current narrative review was conducted to review and highlight treatment of MSD since evidence-based treatment approaches may benefit the patient even years after a brain lesion.^4^ To achieve this objective literature search was conducted using search engines and data bases including google, google scholar, web of science, PubMed from 1998 to 2023 with keywords “motor speech disorder, dysarthria, apraxia, speech motor delay and combinations of these words with English language and no other publication limitations. Our search revealed 170 articles, publications, reports etc., which were downloaded followed by skimming which identified 70 relevant documents of which 34 were used for the review.

## DISCUSSION

Evidence of effectiveness of speech-language therapy in dysarthria is seen following the hierarchy of MSD treatment structure.^4^ The correct treatment approach may benefit the patient even years after a brain lesion.^10^ Different treatment options are employed like Integral stimulation is a term that is designed by Milisen (1956) that elaborates and describes the programs for treatment of articulation. It is one of the imitative methods with emphasis on auditory and visual models.^11^ While planning a rehabilitation program of MSD, it is of utmost importance to teach and apply principles involving medical ethics in the form of code of ethics and conduct as being followed for medical graduates^12^, since SLPs face quite difficult situations while thinking good for the patient at the same time when they are respecting his autonomy and law of land.^13^ Foremost aim of therapy is to get the most out of the efficacy of treatment while enhancing communication resulting in better Qol. Its purpose is to make it more natural and effective during communication, however achieving this requires a lot of effort. The key words that represent the direction of a management goal are to restore, compensate and adjust, which can be elaborated as.^14^

- Functional restoration (restore)
- Promoting the use of residual speech function (compensate)
- Lost function needs reduction (adjust)

Functional restoration is an effort that is made to reduce the speech impairment. Its success is based on the cause and severity of the disease or disorder. Sooner the patient is presented to a SLP with motor speech disorder, the greater will be the chances of his/her recovery. People with reduced physiological abilities of respiration, phonation and articulation need longer periods of therapy then those with less difficulty, while promoting the use of residual speech function that is known as compensation, a phenomenon in which healing of the lost abilities might not get back to normal but the residual abilities can be used to compensate for the one lost. Compensation can be described in many forms for example the use of prosthetic to reduce nasalization or production of certain sound with near accuracy in articulation is also a type of compensation.^14^ Lost function needs reduction means people whose bread and butter is based on their communication abilities are no longer able due to loss of their communication abilities. For improving the lost functions these professionals need to reorganize their life styles and be more focused on practicing vocal hygiene strategies of management.^15^

There are certain factors which influence people who do not address their speech issues in early stages of disease or disorder. With early intervention the probability to be treated with greater accuracy is increased.^5^ However, unfortunately people avoid addressing their problems by avoiding consulting SLPs in a timely manner due to lack of awareness and poor conviction over the fact that therapy can bring positive changes in the wellbeing of an individual with either dysarthria or apraxia. Though, a few general principles are always considered that if an individual is a candidate of treatment or not.^16^ Also dearth of valid, reliable and culturally adequate tools in Urdu language is also a barrier to diagnosis.^17^

The diagnosis of MSD mainly depends on detection of abnormality, impairment or loss of function employing the framework of the World Health Organization’s International Classification of Functioning, Disability and Health (ICF).^18^ Although severity levels of MSD can greatly affect the outcome of management. In general, the management should be designed in a hierarchical manner starting from general to more specific approach while keeping in mind that the communication outcome should reflect upon better Qol. For example, if respiratory support is well established it will help to produce the intelligible speech. Improvement in articulation and nasality will give a better speech output with minimal impairment leading to improved communication.

The duration of therapy depends on the severity and need of a person with MSD. The goals are made for patient to be achieved with the help of clinician and caregiver. Many of the caregivers and patients do not practice the given management techniques prescribed by clinicians and hence, noncompliance leads to delay in goal achievement along with moving towards next target.^19^ There are a wide range of management strategies to treat MSD including multi-disciplinary team as well.^20^ Because of its deviant nature, pathophysiology, characteristics and severity the treatment approach becomes multi-dimensional. Which may include.

- Medical management
- Instrumental and prosthesis
- Behavioral management
- Speech therapy

Unlike apraxia, dysarthria following stroke in which outcome of rehabilitation cannot be predicted,^21^ medical management of childhood MSD’s are known to improve the overall physiological function of an individual as in most cases of stroke or other neurological deficit with poor speech abilities and diagnosed as dysarthria which may vary in severity. Management is not only done by medical specialist alone but a team of specialists like neurologist, otolaryngologist, plastic surgeon and speech pathologist are involved. Some drugs prove to rehabilitate dysarthria’s and apraxia’s in different neurological conditions like Parkinson’s disease, Wilson’s disease, pseudo bulbar palsy and amyotrophic lateral sclerosis.^22^ Prosthetic management is also done as a stop gap arrangement to help patient manage their speech during the process of recovery.

Patient who develops dysarthria or apraxia resulting from accidents may have nasal and/or palatal fractures leading to nasalization or altered speech and managed via palatal prosthesis to let the speech flow normally. Other prosthetic devices are used to modify speech after production like voice amplifiers are used in patients with parkinsonism dysarthria, which increases speech-to-noise ratio, intensity of speech and transcribes intelligibility to supplement communication.^23^ Some other methods are pacing boards, metronomes and delayed auditory feedbacks. There are other tools of behavioral intervention known as augmentative alternative communication (AAC) devices that helps the patient with MSD to use alternative method of communication during their road to recovery by enabling the patient to communicate with the caregivers during treatment leading to less stressful experiences.

It includes picture, word boards letters and other low- and high-tech devices depending on multiple factors like the use of patient, cognitive abilities and socio-economic status.^20^ Behavioral interventions on the other hand, aim to maximize the support for MSD patients to make them to be better able to communicate in the society and positively impacting their Qol. This can be achieved by speaker oriented approach or communication oriented approach. The speaker-oriented approach minimizes the impairment by increasing physiological functions to make it more natural and understandable.

This can be achieved by working on the posture management, improving oro-motor control, strength and range of motion of the articulators to make voice audible and understandable. While, communication-oriented approaches helps to improve communication even if speech is not well developed. It can be done with the help of adding an amplifier to increase loudness level and making it intelligible. It may also be strengthened by decreasing the number of listeners at one time or by adding AAC device to support the communication needs.^24^

Assessment and analysis of dysarthria in neurological diseases is important since it has a greater impact on the psychosocial aspect of life, hence assessment of the condition and its psychosocial effect is important in terms of assessment and management protocol. To achieve this, standardized assessment tools like Dysarthria Impact Profile (DIP) with internal consistency (α = 0.93), concurrent validity (correlation with the VHI: r = -0.77), and discriminant validity (accuracy = 0.93) is essential and tested method 16 hence supports evidence-based practice.

Current practices are moving towards use of advancing technology which is becoming increasingly vital even in the field of speech language pathology, however there is dearth of knowledge among SLPs working across the globe in employing the current trend in their clinical practices. The utilization of technology-based equipment in United Kingdom’s clinical practice differs extensively, and its implementation is hindered by numerous hurdles making it essential to have more collaborative work among SLPs, policy-makers and technologists who develops the foundations of evidence-based practice for technological involvement in rehabilitation of acquired dysarthria as well as phonological delays.^25^

Michell C et al. in a systematic review conducted in 2017 reported that evidence that emerges highlights need for well powered clinical trials, since the studies evaluated, did not reveal benefit and risks of treatments which remains unknown indicating a research gap.^26^ While Whillans C et al. in their systematic review using 14 electronic databases noted 21 studies with 330 cases of dysarthria most with Parkinsonism, taking treatment for acquired dysarthria to determine the evidence of improving production of speech via group therapy with approaches included singing, loudness and multi-components therapies (including combination of impairment and compensatory approaches). Significant improvement was noted for speech production and well-being hence, moderate-quality evidence suggested that group therapy could improve speech and in a number of cases communication and well-being. Singing therapy to some extent even improved intelligibility and Loudness based group therapy was useful in improving intensity measures.^27^ Similarly, another systematic review by Chiaramonte R et al. reported that acoustic parameters which are abnormal in post stroke dysarthria reveal difference after speech therapy interventions.^28^

Speech Motor Delay (SMD), a condition with imprecise, unstable speech, prosody and voice that does not fall into dysarthria or apraxia, is a comparatively newer disorder^9^ and there is need of high-quality research to determine impact of early motor treatment focused on gross motor, locomotor development for infants who are at risk of developing such delay.^29^ However, according to Namasivayam AK et al. for SMD, the PROMPT intervention with at least twice a week for 10 weeks can bring significant results in speech motor control, word level intelligibility and articulation.^30^

With dearth of studies regarding rehabilitation of CAS, a study by Gomez M et al. in 2019 involving online survey among SLPs of Australia and New Zealand revealed that majority of SLPs reported valuing empirical research evidence despite the fact that there are many barriers in the way to rehabilitation of childhood apraxia. Few use eclectic interventions while majority uses Nuffield Dyspraxia Program as their primary intervention. The empirical evaluation of eclectic intervention for childhood apraxia remains unevaluated so far.^31^ While, a study comparing Nuffield Dyspraxia Programme-3 (NDP-3) with Rapid Syllable Transitions Treatment (ReST) for treatment of CAS revealed only limited evidence of improving the accuracy of words in children aged 4-12 years with intensive treatment.^32^

A recent study by Hilary E et al. to determine the accuracy of Treatment for Establishing Motor Program Organization (TEMPOSM) in CAS cases revealed efficacy in improving speech in CAS as regards segmental and supra-segmental impairment.^33^ An Iranian study by Imani-Shakibayi M et al. involving 260 SLPs revealed that though their assessment choice were in line with results of recent studies, however a gap was present as regards knowledge and experience of use of evidence-based treatments since there was a tendency to opt for outdated treatment methods.^34^

Concurrent CD and CAS are associated with lower intelligibility, hence, intelligibility variables should be included in speech-genetic research.^35^ Wilson EM et al did a study in which the main objective was to assess the support of MSD as a descriptive concept to provide guidelines for research and management protocols for intelligibility issues in cases with Down syndrome (DS). Intelligibility status revealed association with classification system for MSD including variables of voice, speech and prosody. 80% of the participants with concurrent childhood dysarthria and CAS evaluated revealed diminished intelligibility which was of low or moderate level while remaining 20% revealed high intelligibility. The participants which met the criteria of having either dysarthria or apraxia had reduced intelligibility effecting communication.^36^

## CONCLUSION

With a wide study on the topic it has been analyzed that there are different treatment and management approaches for MSD that can be utilized in the therapy practices to bring out the best results for the patients. The effect of management strategies depends on the type and severity of motor speech disorders and the practice is highly variable across age groups and different neurological conditions. It has also been observed that therapy or management practice should be speaker oriented and communication oriented to improve the quality of life of people with Motor Speech Disorders.

### Authors Contribution:

**AA:** Literature review and responsible for integrity of the research.

**NM:** Conception and critical revision of article.

**GS:** Review and final preparation of manuscript.
